# “A New Reconstructive Method after Pancreaticoduodenectomy: the Triple Roux on a “P” Loop. Rationale and Radionuclide Scanning Evaluatlon.”

**DOI:** 10.1155/1996/53812

**Published:** 1996

**Authors:** Gianluigi Pescio, Erminio Cariati

**Affiliations:** 1 Surgeon-in-Chief Saint Charles Hospital Bordighera Imperia Italy; 2 Department of Surgery University of Genoa Italy

## Abstract

We propose a method of reconstruction after pancreaticoduodenectomy consisting of a double Roux en Y on the same jejunal loop without interruption of the mesentery and a third anatomical Roux en Y to reconstitute the alimentary tract.

The construction of the double Roux en Y draining pancreas and bile ducts separately, requires a linear Stapler 3-4 centimeters from the biliary anastomosis. In this way, by employing the same loop without mesenteric interruption, two functional excluded loops will be ’obtained. The rationale of the suggested model is based on the separation of biliary and pancreatic secretions. This makes it possible to avoid a stagnant cul-de-sac coinciding with the pancreaticojejunal anastomosis and to obtain in the case of leakage, a pure biliary and/or pancreatic fistula as far as is possible.

99mTc HIDA scans demonstrated the efficiency, of the biliopancreatic limbs of the reconstruction, showing normal emptying time for the gastric remnant and the absence of radionuclide stagnation or any alkaline enterogastric reflux.


*HPB Surgery*, 1996, Vol.9, pp.223–227
Reprints available directly from the publisher
Photocopying permitted by license only

© 1996 OPA (Overseas Publishers Association)
Amsterdam B.V. Published in The Netherlands
by Harwood Academic Publishers GmbH
Printed in Malaysia

### "A New Reconstructive Method after Pancreaticoduodenectomy: the Triple Roux on a "P" Loop. Rationale and Radionuclide Scanning Evaluation."*

[1] GIANLUIGI PESCIO and [2] ERMINIO CARIATI

[1] Surgeon-in-Chief, Saint Charles Hospital, Bordighera, Imperia, Italy
[2] Professor of Surgery and Chairman, Department of Surgery, University of Genoa, Italy.

*(Received 17 March 1994)*

We propose a method of reconstruction after pancreaticoduodenectomy consisting of a double Roux en Y on the same jejunal loop without interruption of the mesentery and a third anatomical Roux en Y to reconstitute the alimentary tract.

The construction of the double Roux en Y draining pancreas and bile ducts separately, requires a linear Stapler 3–4 centimeters from the biliary anastomosis. In this way, by employing the same loop without mesenteric interruption, two functional excluded loops will be obtained. The rationale of the suggested model is based on the separation of biliary and pancreatic secretions. This makes it possible to avoid a stagnant cul-de-sac coinciding with the pancreaticojejunal anastomosis and to obtain in the case of leakage, a pure biliary and/or pancreatic fistula as far as is possible.

99mTc HIDA scans demonstrated the efficiency, of the biliopancreatic limbs of the reconstruction, showing normal emptying time for the gastric remnant and the absence of radionuclide stagnation or any alkaline enterogastric reflux.

KEY WORDS: Carcinoma of the pancreas pancreaticoduodenectomy complications in pancreaticsurgery reconstructive methods after pancreaticoduodenectomy

### INTRODUCTION

Pancreatic fistulae due to leakage of the pancreatojejunostomy after pancreaticoduodenec tomy (PD) may cause severe postoperative complications and death. In the 1960–70's the mortality rate was between 20 and 75–100% [1–5] in the 1980's although the mortality rate was dramatically reduced to < 5%[6–9] and to zero[10] with improved perioperative care, the pancreatic anastomosis continued to be a major cause of morbidity [11,12] and remains around 20% (15%–30%)[10–12].

The introduction of new techniques such as the obliteration of the pancreatic duct with prolamine or fibrin glue [13-21] with or without pancreatojejunostomy, [22,23] improved techniques of anastomosis [24–25] and the revival of the pancreaticogastrostomy [26–28] prove that *the* fistulae [29] still constitutes a serious problem. When leakage at either biliary or pancreatic anastomoses occurs there is a high risk of mortality.

There are various methods which use separate intestinal loops for the pancreatic and biliary anastomoses [30–32] or two separate jejunal segments, one for the biliary and pancreatic anastomosis and another for the gastrojejunostomy to avoid bile reflux into the stomach [33,34].

* The paper is based on a previous communication to the "Updating Course on Pancreatic Diseases", International Meeting, Genoa, April 22-25, 1992 and published as abstract on Hepatogastroenterology, 1992: 47.

*Correspondence to*: Gianluigi Pescio M. D., Via Dardanelli 1/5 16156 Genova, Italia.

In this paper, we propose our reconstructive method which uses three intestinal limbs, with a view to separating the three anastomoses (pancreatic, biliary and gastric) to obtain, in the case of a leak, as pure as possible pancreatic and/or biliary fistula and to avoid alkaline reflux into the stomach.

### MATERIALS AND METHODS-RESULTS

### *A) Technique*

The arrangement consists (fig. 1) of a double Roux en Y on the same jejunal loop, without further mesenteric interruption, draining separately the pancreas and hepatic ducts; for this reason we thought to call it a "functional Roux". The gastrojejunal anastomosis is made by way of a Roux en Y on a second distal loop to restore the alimentary continuity.

The first and proximal jejunal loop has the form of the letter "P", obtained by an end to side jejunal anastomosis on the same loop; the pancreaticojejunal and the biliojejunal anastomosis, are both end to side.

This isolated loop is transposed to the supramesocolic space through the right transverse mesocolon.

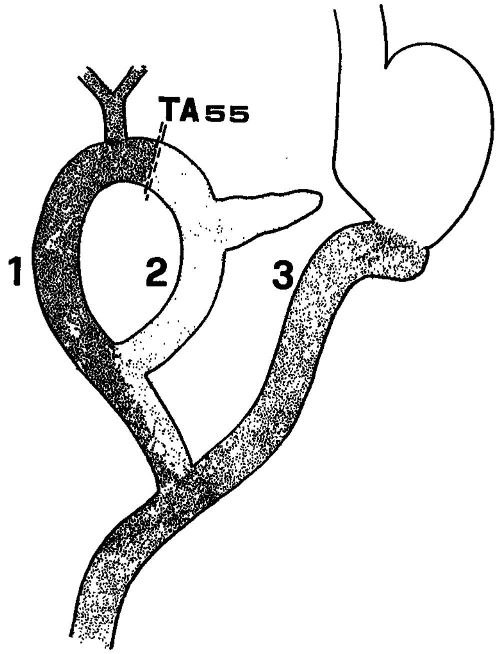

**Figure 1** Reconstruction after pancreaticoduodenectomy using the triple Roux on a "P" loop. The two functional Roux limbs (loop 1 and 2 obtained by a linear stapler TA55) and the true anatomical Roux en Y (loop 3) are shown

For the best placing of the sites of the anastomoses, it may be convenient to carry out the pancreatic anastomosis first, the biliary one as second and finally the jejunojejunal anastomosis. All the anastomoses were made without internal stenting [27] and without ligature or obliteration of the pancreatic duct. [15–22]

### *B) Patients*

We have performed this operation in three cases ([2]carcinoma, [1]chronic pancreatitis): in one of them operated for adenocarcinoma of the pancreas head, without pancreatic duct dilatation and without jaundice, we saw two fistulas, biliary and pancreatic, both of high volume for 10 days.

They were pure, with rock watery liquid from the drainage of the pancreatico-jejunal anastomosis and bile from the subhepatic tube. The patient was discharged on the 30th day after recovery from the survey and enjoyed good health for 8 months with slow but constant recuperation; he died after 18 months.

### *(C) Follow-up studies*

At six and twelve months after discharge, all patients underwent a follow-up by scintiscan to evaluate the morphological and functional aspects of the reconstruction and by metabolic studies to evaluate steatorrhea, malabsorption and malnutrition.

- blood screening included: hematocrit, hemoglobin, bilirubin, gamma GT, serum transaminases, alkaline phosphatase, serum amylase, lipase, glucose, BUN, total serum proteins. Data did not show either megaloblastic anemia or dysproteinaemia.
- determination of fecal fat loss (standard 100 grams fat diet/3 days: loss of more than 7 grams/day is considered abnormal. This was normal in the neoplastic patients, but there was a mean value of 10 grams in the patient with chronic pancreatitis.
- to evaluate intestinal global absorption: D-xylose test, after oral administration of 25 grams and evaluation of the concentration in blood after one and two hours and in the urine after five hours; the normal values in blood are 17% to 12% Normal excretion of D-xylose in the urine is 4,5–7,5 grams. The test was normal in all our patients.
- morphofunctional radionuclide study:

a) 99 mTc HIDA cholescintigraphy [35,36] There was no evidence of bile reflux into the gastric remnant. Scintiscan showed (fig.2,3) the morphology of the reconstruction referring in particular to the linear

Stapler (TA55); we observed a delayed transit time through the "P" loop where the tracer was present for 120 minutes after the test. Moreover HIDA scan showed no bile reflux into the gastric remnant in either patient.

b) gastric emptying study [37–39] after a semisolid radionuclide labelled meal (two eggs, bread and milk 99 mTc mCi; N.V.: T 1/2 = 20'–40'): showed a normal gastric emptying time. Because of our experience ever many years of subtotal gastrectomy with Roux en Y reconstruction without truncal vagotomy, we extend the antrectomy to a subtotal gastrectomy to avoid the Roux en Y syndrome[40–44].

### DISCUSSION

An important point about a reconstruction technique after pancreaticoduodenectomy is the chemical potentiation that can occur with mixing of bile and pancreatic secretions. It is well known[45–47] that both primary bile acids and secondary ones are damaging. They are identified as "detergent–damaging" agents, because of their chemical structure and their presence in bile as conjugated (damaging in acid pH) and non–conjugated (damaging in neutral pH) [46].

The non-conjugated fraction is not very important in the patient after PD, because the absence of the duodenum in which bacterial de-conjugatoion and hydroxylation would take place; but the conjugated fraction remains an effective damaging agent. Biliary lecithin that is hydrolyzed by pancreatic phospholipase A to lysolecithin is a very powerful detergent and caustic agent.

Trypsin has also been the object of studies concerning pancreatic and biliary chemical interaction. The enzyme which is inactive in acid pH and active in neutral pH, may represent another damaging agent among chemical components of alkaline reflux [48]. Other work has shown a relationship between exposure time to damaging agent and the gastric mucosa reaction [49]; this may be important in the chemical-enzymatic digestion of the pancreatic stump by biliary and pancreatic secretions after pancreatic jejunal anastomosis.

The ideal reconstruction after PD, is the triple Roux en Y, pancreatic, biliary and alimentary; but the preparation of three loops has the following dis advantages.

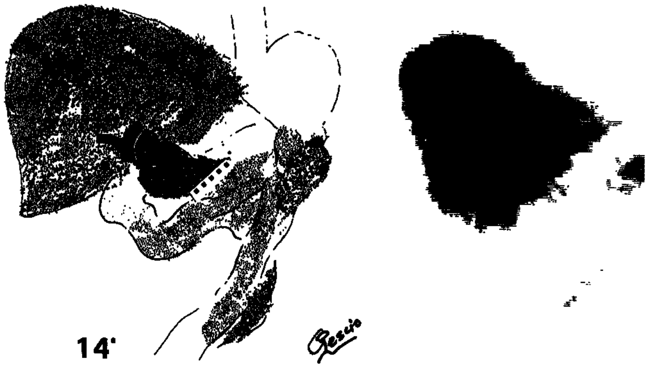

**Figure 2** HIDA 99mTc Cholescintigraphy after 14 minutes: demonstration of the enteric tract between bilio-digestive anastomosis and linear stapler.

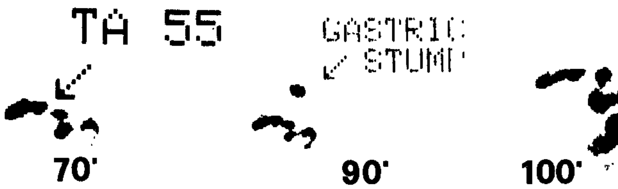

**Figure 3** HIDA 99mTc Cholescintigraphy after 70, 90, 100 minutes: at 70' the linear stapler stop is well demonstrated, at 90' the gastric stump is seen, at 100' the marked liquid bolus shows the alimentary sector of the reconstruction.

- Prolongation of operating time
- increased risk of mesenteric vascular damage;
- more damage to mesenteric innervation with disturbance of peristalsis, with pooling of biliary and pancreatic secretions. The model that we propose is based on these considerations
- no mesenteric and therefore no vascular or nervous interruption to the two loops;
- no cul de sac and no stagnation with dilatation in the proximity of pancreatico–jejunal anastomosis.
- separation between pancreatic and biliary secretions preventing their reciprocal potentiation. A pure pancreatic fistula (with a better prognosis than a mixed one) in the case of a leakage of the pancreatic anastomosis); similarly for the biliodigestive anastomosis;
- The possibility for completion pancreatectomy, when a serious complication requires it
- the "functional double Roux" may be used with or without pyloric preservation.

The morphofunctional radionuclide evaluation proved the efficiency of the biliopancreatic tract, showing a normal emptying time of the gastric remnant and excluding any enterogastric reflux and any radionuclide stagnation [33,34]

According to our preliminary experience, the described technique is useful and indicated for anastomosing the pancreatic stump, when parenchyma is soft and the pancreatic duct is not dilated, factors which often cause an anastomotic leakage. This reconstruction allows a pure fistula without intraluminal pooling; reducing morbidity and mortality: so the recovery time of fistulous complications may be shorter. The third Roux en Y for construction of gastrojejunostomy by duodenal total diversion avoids the incidence of alkaline reflux into the stomach.

In the light of these results we have adopted this reconstruction.

### ACKNOWLEDGEMENTS

We are grateful to Giuseppe Villa, M. D., Service of Nuclear Medicine (Prof. P. Biassoni) of The University of Genoa, for his contribution; and to Alessandro Casano M.D. Our Resident, for his photographic help.

### REFERENCES

1. Monge, J.J., Judd, E.S, Gage, R.D. (1964). Radical pancreatoduodenectomy. A 22-year experience with the complications, mortality rate and survival rate. *Ann. Surg.*, **160**: 711–722.
2. Howard, J.M. (1968). Pancreato'duodenectomy: forty-one consecutive Whipple resections without an operative mortality. *Ann. Surg.*, **168**: 629–636.
3. Gilsdorf, R.B. and Spanos, P. (1973). Factors influencing morbidity and mortality in pancreatoduodenectomy. *Ann. Surg.*, **177**: 332–337.
4. Warren, K.W., Choe, D.S., Plaza, J., Relihan, M. (1975) Results of radical resection for periampullary cancer. *Ann. Surg.*, **181**: 534–550.
5. Sato, T., Saitoh, Y., Notu, N., Matsuno, S. (1977) Follow-up studies of radical resection for pancreatico-duodenal cancer. *Ann. Surg.*, **186**: 581–588.
6. Trede, M., Schwall, G. (1988) The complications of pancreatectomy. *Ann. Surg.*, **207**: 39.
7. Pellegrini, C.A., Heck, C.F., Raper, S., Way, L.W. (1989) An analysis of the reduced morbidity and mortality rates after pancreatico duodenectomy. *Arch. Surg.*, **124**: 778.
8. Ceuterick, M., Gelin, M., Rickaert, F., Van de Stadt, J., Deviere, J., Cremer, M., Lambilliotte, J.P. (1989) Pancreaticoduodenal resection for pancreatic or periampullary tumors: a ten-years experience. *Hepato gastroenterology*, **36**: 467.
9. Peters, J.H., Carey, L.C. (1991) Historical review of pancreaticoduodenectomy. *Am. J. Surg.*, **161**: 219–225.
10. Trede, M., Schwall, G., Saeger, H.D. (1990) Survival after pancreatoduodenectomy: 118 consecutive resections without an operative mortality. *Ann. Surg.*, **211**: 447.
11. Van Heerden, J.A, (1984) Pancreatic resection for carcinoma of the pancreas. World *J. Surg.*, **8**: 880–888.
12. Smith, C.D., Sarr, M.G., Van Heerden, J.A. (1992) Completion pancreatectomy following pancreaticoduodenectomy: clinic experience. World *J. Surg.*, **16**: 521–524.
13. Whittrin, G., Jost, J., Clemens, M., Arndt, M. (1981) Pankreasgangocclusion nach partieller Duodenopankreatektomie in der Carcinomtherapie. *Chirurg*, **52**: 157–159.
14. Gebhardt, C.H., Stolte, M. (1982) Pankreasgangocclusion-Mogliche fehler und Fehlinterpretationen. *Chirurg*, **53**: 325–331.
15. Chapuis, Y., Yandza, T., Bonnichon, Ph., Delaitre, B., Grateau, F. (1987) L'exclusion de l'anastomose pancréato-jéjunale réduit-elle la mortalité de la duodénopancréatectomie céphalique? *Chirurgie*, **113**: 262–269.
16. Gossot, D., Gallot, D., Baudot, H., Seleur, A., Malafosse, M. (1988) Les suppurations après obturation du canal de Wirsung par une solution de Prolaminacide (Ethibloc). *J. Chir.*, **125**: 674–675.
17. Giuly, J., Francois, G.F. (1989) L'emploie de la prolaminacide (Ethibloc) reduit-il la mortalité de la duodéno-pancréatectomie céphalique? *J. Chir.*, **126**: 421.
18. Kram, H.B., Clark, S.R., Ocampo, H.P., Yamaguchi, M.A., Shoemaker, W.C. (1991)Fibrin glue sealing of pancreatic injuries, resection and anastomoses. *Am. J. Surg.*, **161**: 479–81.
19. Cavallini, M., Tallerini, A., Stipa, F. (1991) Occlusione del dotto con colla di fibrina e conservazione del piloro dopo duodenocefalopancreasectomia per carcinoma ampollare. *Min. Chir.*, **46**: 733–739.
20. Di Carlo, V., Carlucci, M., Staudacher, C., Zerbi, A., Braga, M., Cristallo, M. (1992) Neoprene injection into the Wirsung duct after pancreatoduodenectomy instead of pancreatojejunal anastomosis. *Chirurgia*, **5**: 136–139.
21. Di Carlo, V., Chiesa, R., Pontiroli, A.E. (1984) Intraductal injection of Neoprene to suppress native pancreatic exocrine secretion in humans: clinical and metabolic evaluation. *Transpl. Proc.*, **16**: 736–738.
22. Goldsmith, H.S., Ghosh, B.C., Huvos, A.G. (1971) Ligation versus implantation of the pancreatic duct after pancreatoduodenectomy *Surg. Gynecol. Obstet.*, **133**: 87–92.
23. Gazzaniga, G.M., Pastorino, G., Castagnola, M. (1992) Treatment of the pancreatic stump after pancreatoduodenectomy with pancreatojejunal anastomosis. Chirurgia, **5**: 140–144.

24. Kingnorth,A.N. (1989)Duct to mucosa Roux loop pancreato-jejunostomy as an improved anastomosis after resection of the pancreas. *Surg. Gynecol. Obstet.*, **169**: 451–453.
25. Biehl, T., Traverso, L.W. (1992) Is stenting necessary for a successsful pancreatic anastomosis? Am. *J. Surg.*, **162**: 530–532.
26. Millbourn, E. (1958) Pancreatico-gastrostomy in pancreatico-duodenal resection for carcinoma of the head of the pancreas or the papilla of *Vater. Acta Chi. Scand.*, **116**: 12–17.
27. Park, C.D., Mackie, J.A., Rhoads, J.E. (1967) Pancreaticogastrostomy. *Am. J. Surg.*, **113**: 85–89.
28. Telford, G.L., Mason, G.R. (1981) An Improved technique for pancreati-cogastrostomy after pancreaticoduodenectomy. *Am. J. Surg.*, **142**: 386–87.
29. Frey, C.F. (1992) Pancreatic fistulas and tumors of the pancreas. In: Pederzoli, P., ed. Pancreatic Fistulas. pag. 85–86, Berlin Heidelberg: Springer-Verlag.
30. Machado, M.C.C., Moteiro da Cunha, J.E., Bacchella, T., Bove, Raia, A. (1976) A modified technique for reconstruction of the alimentary tract after pancreatoduodenectomy. *Surg. Gynecol. Obstet.*, **143**: 271–273.
31. Funovics, J.M., Zoch, G., Wenzl, E., Schulz, F. (1987) Progress in reconstruction after resection of the head of the pancreas. *Surg. Gynecol. Obstet.*, **164**: 545–548.
32. Gozzetti, G., Frena, A., Principe, A., Fuga, G. (1994) Experience with a personal technique for reconstruction after pancreaticoduodenectomy. In: Gazzaniga, G.M. ed. What's new on pancreatic diseases pag. 191–193, Stuttgart. New York: Georg Thieme Verlag.
33. Lygidakis, N.J., Brummelkamp, W.H. (1985) A new approach for the reconstruction of continuity of the alimentary tract after pancreaticoduodenectomy. *Surg. Gynecol. Obstet.*, **160**: 453–458.
34. Lygidakis, N.J., Van der Hyde, M.N., Houthoff, H.J. *et al.* (1989) Resectional surgical procedures for carcinoma of the head of the pancreas. *Surg. Gynecol. Obstet.*, **168**: 157–165.
35. Tolin, R.D., Malmud, L.S., Stelzer, F. (1979) Enterorgastric reflux in normal subjects and patients with Billroth-II gastroenterostomy. Gastroenterology, **77**: 1027–1032.
36. Harvey, E., Loberg, M., Cooper, M. (1975) 99mTc HIDA. A new radiopharmaceutical for hepatobiliary imaging. *J. Nucl. Med.*, **16**: 533.
37. Harvey, E., Brown, J.J.G., Mackie, D.B., Keelintg, D.H. (1970) Measurement of gastric emptying time with a gamma camera. Lancet, **1**: 16–18.
38. Patti, M.G., Pellegrini C.A., Way, L.W. (1987) Gastric emptying and small bowell transit of solid food after pylorus-preserving pancreaticoduodenectomy. *Arch. Surg.*, **122**: 528–532.
39. Linehan, I.P., Russel, R.C.G., Hobsley, M. (1988) The dumping syndrome after pancreatoduodenectomy. *Surg. Gynecol. Obstet.*, **167**: 114–118.
40. Vogel, S.B., Hocking, M.B, Woodward, E.R. (1983) Pre and postoperative radionuclide gastric emptying evaluation in patients undergoing Roux-en-Y biliary diversion for alkaline reflux gastritis and postgastrectomy dumping. *Surg. Forum*, **34**: 173.
41. Donahue, P.E., Bombeck. C.T., Condon, R.E., Nyhus, L.M. (1984) Proximal gastric vagotomy versus selective vagotomy with antrectomy: result of a prospective randomized clinical trial after four to twelve years. *Surgery*, **96**: 585–591.
42. Nyhus, L.M., Wastell, C. (1986) Special problems. In: *Surgery of the stomach and duodenum*. Vol. V, Pag. **419**. Boston/ Toronto: Brown and Co.
43. Ehrlein, H.J., Buhner, S., Thoma, G. (1987) Gastric emptying after Roux-en-Y and Billroth-I gastrectomy depends on viscosity of the meal and contractile patterns of small intestine in dogs. Dig. Dis. Sci., **32**: 529–537.
44. Buhner, S., Ehrlein, H.J., Thoma, G., Schumpelick, V. (1988) Canine motility and gastric emptying after subtotal gastrectomy. *Am. J. Surg*. **156**: 196–199.
45. Davenport, H.W. (1970) Effect of lysolecithin, digitonin and phospholipase A upon the dog's gastric mucosal barrier. Gastroenterology **59**: 505–509.
46. Ivey, K.J., Denbesten, L., Clifton, J.A. (1970) Effect of bile salts on ionic movement across the human gastric mucosa. Gastroenterology **59**: 683.
47. Gadacz, T.R., Zuidema, G.D. (1978) Bile acid composition in patients with and without symptoms of postoperative reflux gastritis. *Am. J. Surg.*, **135**: 48.
48. Salo, J.A., Kivilaakso, E. (1984) Contribution of trypsin and cholate to the pathogenesis of experimental alkaline reflux esophagitis. *Scand. J. Gastroenterol.*, **19**: 375.
49. Lawson, H.H. (1981) Effect of duodenal contents on the gastric mucosa under experimental conditions. *Scand. J. Gastroenterol.*, **16** (Suppl 67) 91.

